# App-Based Addiction Prevention at German Vocational Schools: Implementation and Reach for a Cluster-Randomized Controlled Trial

**DOI:** 10.1007/s11121-024-01702-w

**Published:** 2024-07-03

**Authors:** Diana Guertler, Dominic Bläsing, Anne Moehring, Christian Meyer, Dominique Brandt, Hannah Schmidt, Florian Rehbein, Merten Neumann, Arne Dreißigacker, Anja Bischof, Gallus Bischof, Svenja Sürig, Lisa Hohls, Maximilian Hagspiel, Susanne Wurm, Severin Haug, Hans-Jürgen Rumpf

**Affiliations:** 1 Institute for Community Medicine, Department of Prevention Research and Social Medicine, University Medicine Greifswald, Walther-Rathenau-Str. 48, 17475 Greifswald, Germany; 2 Institute for Community Medicine, Department of Methods in Community Medicine, University Medicine Greifswald, Walther-Rathenau-Str. 48, 17475 Greifswald, Germany; 3 DZHK (German Centre for Cardiovascular Research), Partner Site Greifswald, Greifswald, Germany; 4 Helmholtz Institute for One Health, Greifswald, Germany; 5 Department of Psychiatry and Psychotherapy, University of Lübeck, Lübeck, Germany; 6 Department of Social Work, FH Münster University of Applied Sciences, Muenster, Germany; 7 Criminological Research Institute of Lower Saxony (KFN), Hannover, Germany; 8 Swiss Research Institute for Public Health and Addiction, University of Zurich, Zurich, Switzerland

**Keywords:** Addiction, Prevention, Mobile app, Vocational student, Reach

## Abstract

This article examines the implementation, participation rates, and potential determinants of participation in the digital addiction prevention program “ready4life.” A two-arm cluster-randomized trial recruited German vocational students via class-based strategies. Intervention group received 16 weeks of in-app coaching; the control group received health behavior information, with coaching offered after 12 months. Potential determinants of participation were analyzed based on class and individual characteristics. Out of 525 contacted schools, 35 participated, enrolling 376 classes. Implementation during the pandemic required flexible adjustments, with 49.7% of introductions conducted in person, 43.1% digitally via online streaming, and 7.2% received a video link via email. Despite challenges, 72.3% of the vocational students downloaded the app, and 46.7% gave informed consent. Participation rates were highest among (associate) professionals, vocational grammar school classes, classes introduced by females, younger individuals, members of the project team, and classes introduced face-to-face. Female gender, lower social competencies, lifetime cannabis use, higher problematic internet use, and higher perceived stress were associated with higher individual participation. The study highlights the importance of proactive outreach and personalized interventions for addiction prevention programs in vocational schools. While reached students aligned with the aims of the app, tailored recruitment strategies could enhance engagement among under-represented groups. The trial was registered in the German Clinical Trials Register (DRKS): DRKS00022328; registration date 09.10.2020.

**Supplementary Information:**

The online version contains supplementary material available at 10.1007/s11121-024-01702-w.

Vocational students show elevated rates of substance use. For example, among vocational students in Switzerland, 52.1% reported problematic alcohol consumption, 44.0% smoking of tobacco or nicotine products (including cigarettes, shisha, cigars, e-cigarettes), and 28.2% cannabis consumption in the last 6 months Paz Castro et al. (2023). Similar rates have been reported from representative data of German vocational students in Mecklenburg-Western Pomerania (Meyer et al., 2016). Moreover, a substantial proportion of vocational students report problematic internet use (75.2%) (Paz Castro et al., 2023). Although many vocational students use the internet without any negative consequences, it becomes problematic when it starts to interfere with daily life (e.g., academic performance, relationships, or responsibilities) or leads to negative consequences (e.g., losing sleep). This can happen if they spend excessive amounts of time online, use the internet to escape negative moods, or experience difficulties in controlling their internet use.

Many vocational students engage in multiple of the above-mentioned behaviors and have low intentions to change (Atorkey et al., 2021a, 2022; Meyer et al., 2016), predisposing them to noncommunicable diseases. Being in the developmental and explorative stage of adolescents or young adulthood, vocational students may prioritize immediate gratification and social acceptance over long-term consequences, making it harder for them to recognize the need for change (Atorkey et al., 2021b). Peers and vocational settings further perpetuate this cycle of behavior due to prevailing positive consumption norms (Saetta et al., 2023; Trucco, 2020). Studies suggest that vocational students who struggle with social competencies like approaching others, expressing needs, or resisting group pressure are more likely to experience probleminternet and alcohol use (Paz Castro et al., 2023). Moreover, vocational students may resort to substance or internet use as coping mechanisms for stress (e.g., Gioia et al., 2021). This is supported by data showing that stress is associated with higher substance and internet use in vocational students (Paz Castro et al., 2023). Particularly, polysubstance use has been linked to lower social competencies (de Jonge et al., 2022) and higher job-related stress (Tomczyk et al., 2016). Consequently, interventions within the vocational school setting should adopt a comprehensive approach, targeting multiple behaviors such as substance and internet use. Simultaneously, they should foster essential life skills, including social competencies and stress regulation, to equip vocational students with the tools necessary for healthier decision-making and coping mechanisms.

Digital interventions (Aneni et al., 2023; Monarque et al., 2023) and chatbots providing personalized feedback (Ogilvie et al., 2022; Przewoźniak et al., 2021) have been used effectively to change substance use among adolescents and (young) adults. Especially mobile technology can enhance implementation and effectiveness by addressing time and cost barriers, offering cost-effective, personalized content delivery, and anonymous participation (Kazemi et al., 2021). Reach, the proportion and representativeness of users, significantly influences the population-level effect of digital interventions (Holtrop et al., 2021). On the intervention level, two characteristics may play a crucial role in the enhancement of reach: proactive delivery and tailoring. Proactive delivery, offering interventions during school hours to all students independent of their shown risk profile, expands proportion and representativeness (Velicer et al., 2000), particularly for those not yet planning to change their behavior. Previous programs within the school setting have reached 59–80% of eligible students using this approach (Haug et al., 2023a, b; Paz Castro et al., 2021). Tailoring of intervention content to the needs of students is crucial as they largely differ in their individual risk profile (Meyer et al., 2016). In multi-behavior interventions, allowing users to self-select intervention content (e.g., modules related to specific substances) is an enhanced form of tailoring and boosts user autonomy (Ryan & Deci, 2000). This choice-driven approach can amplify motivation, self-efficacy, and commitment, while countering resistance. Given that many vocational students do not currently plan to change their substance or internet use (Meyer et al., 2016), their willingness for substance-specific prevention programs might be limited. Introducing options for self-selection of non-substance-specific life skills such as social competencies or dealing with stress could enhance their participation willingness. This is supported by evidence showing that life skills promotion programs achieve higher participation rates (82–84%) than substance-specific trainings (50–75%) (Paz Castro et al., 2021).

Only two studies in vocational settings evaluated app-based multi-behavior interventions (Haug et al., 2022; Pietsch et al., 2023). Pietsch et al. (2023) addressed tobacco, e-cigarettes, alcohol, and cannabis use as well as gambling and digital media–related behaviors using a voluntary commitment approach to reduce or abstain from one of these behaviors. Haug et al. (2022) used a chatbot to provide feedback on two self-selected behaviors, including substance use (tobacco, alcohol, cannabis), problematic internet use, or life skills. Both studies demonstrated the feasibility and effectiveness of such interventions in the vocational school context, with largest reductions in at-risk alcohol use, and problematic internet or social media use.

Beyond intervention characteristics, individual attributes influence reach. The yet scarce evidence in terms of digital addiction prevention programs within the (vocational) school setting suggests that higher problem severity, medium (vs. low) level of stress, higher intention to change, female gender, and younger age are associated with participation (Haug et al., 2023b; Haug & Castro, 2018; Haug et al., 2015; Paz Castro et al., 2021; Schmid et al., 2015).

## Aim

The “ready4life” digital prevention program, originally developed in Switzerland (Haug et al., 2017, 2020, 2022), addresses substance use, problematic internet use, and life skills in vocational students. It employs a facilitated access approach through class-based proactive recruitment. Students could choose two out of six intervention modules to be individually coached. This study aims to (1) describe the program’s implementation in German vocational schools (Schmidt et al., 2023) during the COVID-19 pandemic, (2) assess student participation, and (3) identify class and individual characteristics associated with participation. We were particularly interested in whether the participation factors were in line with the aims of the intervention, e.g., reaching students with high levels of stress and low social competencies.

## Material and Methods

### Study Design

The “ready4life” program was evaluated among German vocational students aged 16 and older in a two-arm cluster-randomized design (Puffer et al., 2003). Clusters comprised classes from vocational schools across five German federal states. Follow-up assessments occurred 6 and 12 months after the baseline and were conducted via online surveys. The primary outcome was change in substance use (alcohol, tobacco, cannabis) and problematic internet use at the individual level. The trial protocol was previously documented (Schmidt et al., 2023).

### Participants and Procedure

Approval from ministries for education and cultural affairs in each German federal state was secured prior to recruiting vocational schools. Once permission was granted, schools were initially contacted via email, followed by telephone communication. Participating schools registered through an online platform. The study’s implementation in vocational schools followed these steps: **Step 1**: School administrators or teachers selected classes to be enrolled. Project or school staff registered classes online. To mitigate selection bias, class selection occurred prior to randomization. No eligibility criteria were applied at class level. On individual level, vocational students, 16+, with smartphone and contact (email/phone) were eligible for study participation. **Step 2**: After registration, classes were randomly allocated to intervention or control condition stratified by school and in blocks of four (computer generated with allocation ratio of 1:1). Class-specific passwords were created, which determined the assignment to the study group (intervention or control group). **Step 3**: The app was introduced to all students during school hours. Vocational students received detailed information about the study, including funding, purpose, procedures, randomization, incentives, data protection, and the app’s features like content and functions, along with a raffle. To maximize reach, it was emphasized that students could freely choose modules. Introductions took place between October 2020 and March 2022. Recruitment coincided with restrictions imposed by the COVID-19 pandemic; thus, various adjustments became necessary to program implementation (see Online Resource 1). Introduction were either face-to-face, via online streaming, or an emailed YouTube video link was used. **Step 4**: Students downloaded the app, and to choose either a male or female digital coach. **Step 5**: Students then independently completed an anonymous app-based screening covering topics such as alcohol, tobacco, and cannabis consumption, use, social competencies, and stress. **Step 6**: Digital informed consent was collected from individual vocational students via the app (Online Resource 1). Additional parental consent was not collected. Underaged participants were advised that their parents or legal guardians must be informed about study participation. For this purpose, a link to a digital information letter was provided. **Step 7**: Students received feedback on their individualized risk and competence profile in form of a traffic light system for each assessed topic (Online Resource 2). Topic-specific thresholds for receiving a green, yellow, or red traffic light are displayed in Online Resource 3. Students were then informed about their group assignment. **Step 8**: Intervention group participants chose two out of six modules and received 16 weeks of coaching via the “ready4life” app, described below. Control group participants received a link to information on enhancing health behaviors and could access the coaching after 12 months. **Step 9**: Students were invited to two follow-up sessions at 6 and 12 months via SMS or email with a link to an online-based chat conversation with the digital coach. Participants who did not complete the follow-up were reminded by SMS or email, and were contacted by telephone. **Step 10**: Vouchers were raffled among participants.

### The “ready4life” Program

The app-based coaching program “ready4life,” originally developed in Switzerland (Haug et al., 2020), aims to prevent or reduce substance use and problematic internet use, and promote life skills in vocational students. The app version (Schmidt et al., 2023) used in this study included an app-based screening and individualized feedback (risk and competence profile). After the screening, students could choose two out of six modules: alcohol, tobacco, cannabis, social media/gaming, stress management, or social competencies. Within each module, 8 weeks of coaching were provided. The two selected modules were offered in a random order. Coaching involved weekly 5-min chats with a conversational agent (chatbot), incorporating media (videos, images, links) and allowing user-initiated dialogues. Chats followed predefined rules and did not utilized artificial intelligence. To improve adherence (Jakob et al., 2022), the app included tailored content, push notifications, social and gamification features (quizzes, contests, collecting credit points based on completing the weekly dialogue, prize winning), and personal support in form of an “ask-the-expert” function. The “ready4life” app is free, ad-free, and without in-app purchases. Refer to Online Resource 2 for app screenshots. Students indicating any need for intervention from professionals (e.g., mentioning suicidal ideation or other crises during the asked “ask-the-expert” function) were provided with contact details of a local, free 24/7-telephone counselling service. Within the app, students’ contact details (email, phone number) were collected for follow-up data collection and information on prize winning. Research data collected via the app was stored pseudonymized and separate from contact details.

### Measurement

#### Class Level

The following data were collected during class registration: federal state of school, the classes’ educational track, years of education, class size, number of students present during introduction, mode of introduction, introduction time, introducing person, age, and gender of introducing person as well as if the introduction was held in a tandem, i.e., with two introducing persons. For students in vocational training, the International Standard Classification of Occupations 2008 (ISCO-08) was used to classify occupations (Online Resource 4).

#### Individual Level

Individual data were assessed self-administered within the app-based screening.

##### Socio-demographics

Age (calculated from the provided date of birth) and gender.

##### Alcohol Consumption

Questions based on the Alcohol Use Disorders Identification Test - Consumption (AUDIT-C) (Bush et al., 1998). Students reported alcohol use frequency in the past 30 days (0–30 days). If any use was reported, details on beverage type and number of drinks on a typical day were collected. A digital bar showed common serving sizes (e.g., 0.5 l beer, 2 cl shot). Using chosen drinks, standard drinks were calculated (1 standard drink = 12 g alcohol). A quantity-frequency index was computed with drinking days multiplied by typical standard drinks per day divided by 30.

##### Tobacco Consumption

The frequency of tobacco smoking (cigarettes, shisha, cigars, etc.) or nicotine product use (e-cigarette, e-shisha, etc.) in the last 30 days was assessed. Response options included 1 “(almost) daily,” 2 “occasionally but not daily,” or 3 “never.” For those who reported any smoking, the number of consumption days (0–30 days) and cigarettes smoked per day within the last month were assessed. We computed a quantity-frequency index by multiplying consumption days by daily cigarettes smoked, divided by 30.

##### Cannabis Consumption

Lifetime consumption of THC-containing cannabis was assessed with the response options 1 “no, never” and 2 “yes.” For individuals who reported ever consuming THC-containing cannabis, the frequency of consumption in the last 6 months was assessed using the following response options: 0 “not at all,” 1 “once a month or less,” 2 “2-4 times a month,” 3 “2-3 times a week,” 4 ”4 times a week or more.” Additionally, the number of consumption days in the last month (0–30 days) was collected.

##### Problematic Internet Use

Problematic internet use was assessed using the Short version of the Compulsive Internet Use Scale (Short CIUS) (Besser et al., 2017). It comprises five items, e.g., “How often do you find it difficult to stop using the internet when you are online?” or “How often do you neglect your everyday commitments because you prefer to go online?,” with response options: 0 “never,” 1 “rarely,” 2 “sometimes,” 3 “often,” and 4 “very often.” A total sum score was calculated, with a possible range of 0 to 20. Sum scores of ≥ 7 indicate a problematic use of the internet (Besser et al., 2017). Cronbach’s alpha was 0.76, comparable to the 0.77 reported by Besser et al. (2017).

##### Perceived Stress

Stress was assessed by one question (Elo et al., 2003): “Stress is a state in which a person feels tense, restless, nervous, or anxious, or is unable to sleep at night due to disturbing thoughts. How much do you currently feel this type of stress?” (5-point Likert scale from 1 “not at all” to 5 “very strongly”). Stress was treated as continuous.

##### Social Competencies

Eight items based on the assertion inventory (Gambrill & Richey, 1975) were used. Two items each addressed the following aspects: (1) approaching others, (2) expressing needs, (3) enduring group pressure, and (4) standing up for oneself. Answer options included 1 “very uncertain,” 2 “rather uncertain,” 3 “mixed/neutral,” 4 “certain,” and 5 “very certain”. A total sum score was calculated (possible range: 8 to 40). Cronbach’s alpha was 0.75, higher than the 0.65 reported previously in vocational students (Haug et al., 2017).

##### General Self-efficacy

A short scale for measuring general self-efficacy was used (ASKU) (Beierlein et al., 2012) which included three items (“I can rely on my own abilities in difficult situations.”, “I am able to solve most problems on my own.”, “I can usually solve even challenging and complex tasks well.”) answered on a 5-point Likert scale (1 “doesn't apply at all,” 2 “applies a bit,” 3 “applies somewhat,” 4 “applies mostly,” 5 “applies completely”). A total sum score was calculated (possible range: 3 to 15). Cronbach’s alpha was 0.79, similar to the 0.80 reported by Décieux et al. (2019) in adolescents.

##### Participation

Each download of the app was logged with the used class password. Study participation was defined as giving informed consent and deposited contact details for follow-up assessment.

### Data Analysis

The number of enrolled states, schools, and classes was reported. The enrolled classes were described in terms of class level variables listed above. Downloads and participation rates were calculated at class and individual level by dividing the number of study participants by the number of students that were present during app introduction. Potential determinants of class level participation rate were analyzed by using linear regression analyses. These determinants included the class level variables listed above. For individual level participation (coded as declined participation vs. participated), logistic multilevel regression analyses with a random intercept on class level (Twisk, 2006) were used to account for the clustered data structure. Intra-Class Correlation (ICC) was computed using an intercept-only model. ICCs express the percentage of total variance in participation that is attributed to the cluster variable (e.g., classes) (Twisk, 2006). Both class level and individual level variables listed above were examined. Regression coefficients or odds ratios were reported along with confidence intervals (CIs) and *p*-values. Potential determinants were described using *n* and percentages, means and standard deviation (SD), or median and interquartile range (IQR) were appropriate. Mean participation rates were reported for each level of categorical variables. All analyses were performed univariate using Stata/Se 17.0.

## Results

### Enrolled States, Schools, and Classes

**Fig. 1:**
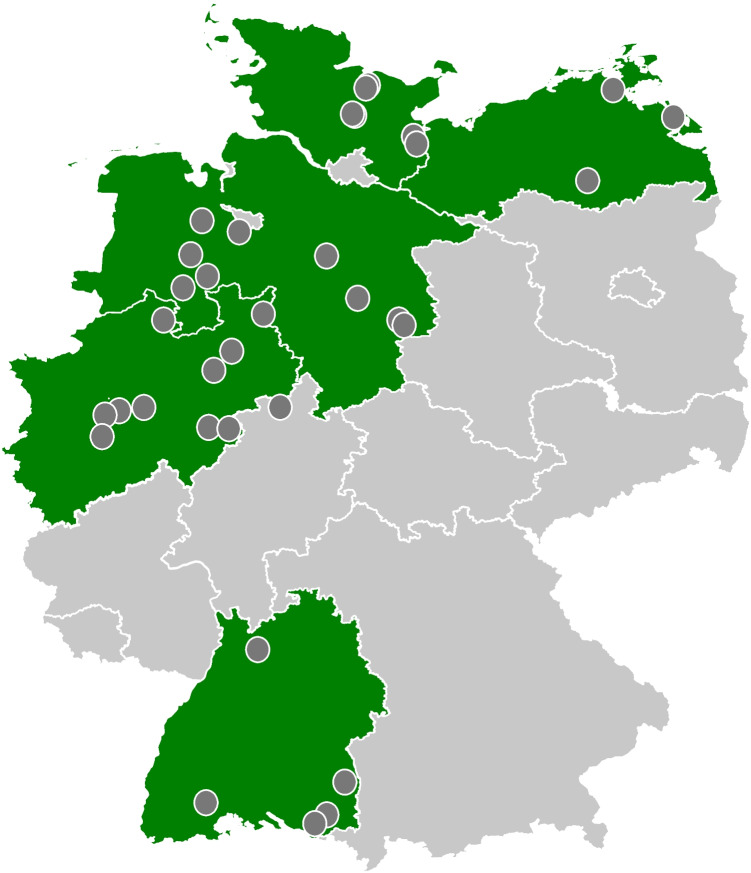
Location of participating schools across Germany

For five federal states, permission for recruitment of vocational schools was received (Table 1). Reasons for not receiving permission for recruitment are included in Online Resource 5. In total, 525 schools were contacted and invited for study participation. Of them, 35 (6.7%) participated in the study (Fig. 1). In total, 376 classes were enrolled in the study (M = 10.7 classes per school, SD = 12.9, range 1–54). The largest proportion of enrolled classes originated from Schleswig-Holstein (35.9%) and Niedersachsen (27.9%) and were enrolled in traditional vocational training programs (64.9%; Table 1). Most apprentices were in their first (40.6%) or second (40.6%) year of vocational training. On average, class sizes were 17.9 students (SD = 6.6, range = 3-47). On the day of the introduction in the classes, an average of 15.7 students were present (SD = 6.5, range = 2-44).

**Table 1:** Description of enrolled classes (*n* = 376)

**Federal state, *n* (%)**	Baden-Württemberg	41 (10.9%)
	Mecklenburg-Vorpommern	22 (5.9%)
	Niedersachsen	105 (27.9%)
	Nordrhein-Westfalen	73 (19.4%)
	Schleswig-Holstein	135 (35.9%)
**Study group, *n* (%)**	Intervention	186 (49.5%)
	Control	190 (50.5%)
**Educational track, *n* (%)**^a,b^	Vocational training	244 (64.9%)
	Professionals	3 (0.8%)
	Technicians and associate professionals	59 (15.7%)
	Clerical support workers	41 (10.9%)
	Service and sales workers	54 (14.4%)
	Craft related trades workers	68 (18.1%)
	Plant and machine operators and assemblers	11 (2.9%)
	Elementary occupations	1 (0.3%)
	Mixed occupations	7 (1.9%)
	Vocational grammar school^g^	77 (20.5%)
	Vocational preparation^h^	48 (12.8%)
**Year of education, *n* (%)**^a,c^	First year	157 (41.8%)
	Second year	122 (32.5%)
	Third year	35 (9.3%)
**Students in class, M (SD)**^d^		17.9 (6.6)
**Present students, M (SD)**^e^		15.7 (6.5)
**Mode of introduction, *n* (%)**	Digitally/hybrid	162 (43.1%)
	Face-to-face	187 (49.7%)
	Email	27 (7.2%)
**Time for introduction, *n* (%)**^a,f^	45 min	275 (73.1%)
	70–90 min	68 (18.1%)
**Introduction by, *n* (%)**	Members of the project team	239 (63.6%)
	Schoolteachers	56 (14.9%)
	Addiction prevention experts	34 (9.0%)
	School social workers	22 (5.9%)
	Health education students	25 (6.7%)
**Gender of IP, *n* (%)**	Male	107 (28.5%)
	Female	269 (71.5%)
**Age of IP, M (SD)**		30.2 (8.3)
**Tandem introduction, *n* (%)**	No	327 (87.0%)
	Yes	49 (13.0%)

*IP* introducing person, *M* mean, *SD* Standard deviation

^a^percentages do not add up to 100 due to missing information

^b^information is missing for *n* = 4 (1.1%) classes and *n* = 3 (0.8%) classes included students from different educational tracks

^c^information is missing for *n* = 37 (9.8%) classes and *n* = 25 (6.7%) classes included students from different years of education

^d^information is missing for *n* = 10 (2.7%) classes

^e^information is missing for *n* = 13 (3.5%) classes

^f^information is missing for *n* = 6 (1.6%) of classes and *n* = 27 (7.2%) classes were introduced by email

^g^in Germany, most vocational schools also offer participation in vocational grammar school classes (typically grades 11 to 13) to prepare students for general university entrance certification

^h^these include vocational preparation classes as well as 1- or 2-year basic training with intermediate secondary school-leaving certificate (without training qualification)

### Implementation of Introductions

Despite the challenging conditions during the pandemic, half (49.7%) of all classes were introduced to the project in person. Another 43.1% were introduced digitally or in a hybrid form, and a further 7.2% were introduced exclusively via email. The typical duration of introductions was 45 min (73.1% of introductions). Introductions were mainly conducted by members of the project team (63.6%), followed by trained schoolteachers (14.9%), addiction prevention experts (9.0%), trained school social workers (5.9%), or trained vocational students (6.7%). The introductions were predominantly conducted by females (71.5%) with an average age of 30.2 years (SD = 8.3). In 13.0% of the introductions, a second person was present (81.6% of whom were female, with an average age of 26.8 years, SD = 11.4).

### Study Participation of Vocational Students

**Fig. 2:**
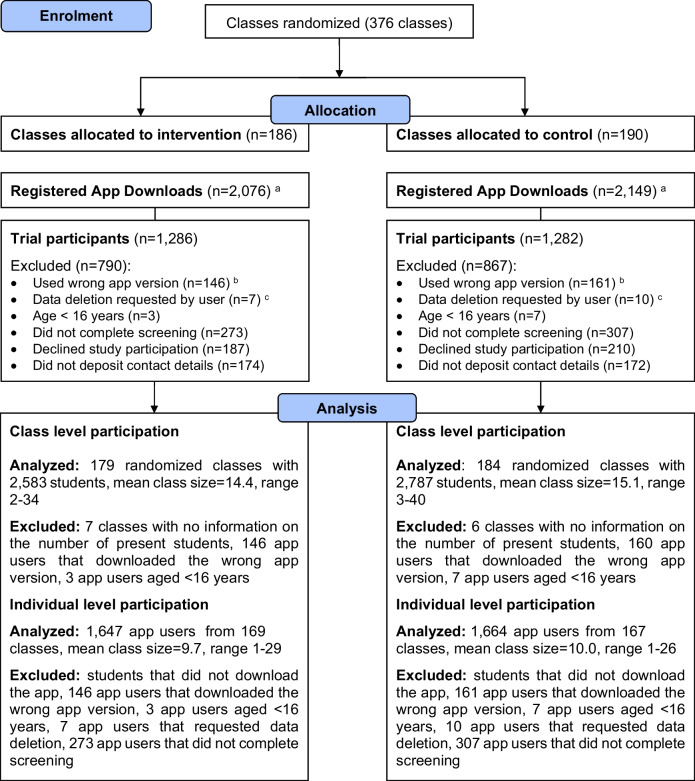
Flowchart of clusters and participants. Note.^a^*n* = 11 students were identified who had registered for the study twice; these have already been subtracted from the download count; ^b^due to technical reasons, *n* = 307 students used a different app version than that one being tested; in consequence of this, informed consent for the current study was not obtained from these students; ^c^app users were able to initiate the deletion of all data collected at any time-point regardless of whether they just started the screening or provided informed consent and were using the intervention already

Out of 376 classes, 186 were assigned to the intervention group, and 190 to the control group (Fig. 2). The app was downloaded 4225 times. However, 307 students received a different app version due to a technical error. The error was assumed to be random as it was not region or school specific. Additionally, 17 students requested data deletion via email and 10 students were under the age of 16. From the remaining students, 2568 provided consent and contact details for follow-up. Calculations for download and participation rates were based on classes with information on the number of students present during the introduction (96.5%, *n* = 363 classes with a total of 5686 students). For participation rates, students who had downloaded the wrong app version (*n* = 306 students within the 363 classes) and app users under the age of 16 (*n* = 10 students within the 363 classes) were considered neutral dropouts. On the class level, download and participation rates varied between 0 and 100% across classes with a mean download rate of 69.3% (SD = 0.33) and a mean participation rate of 46.7% (SD = 0.31). On the individual level, the download rate was 72.3% (4113 of 5686 present students) and participation rate was 46.7% (2508 of 5370 present students, aged 16+, with the correct app version).

### Potential Determinants of Participation on the Level of the School Class

Participation rates of classes differed according to their federal state, educational track, class size, and the mode and time of introduction as well as the characteristics of the introducing person (Table 2). The highest participation rates were observed for classes from Schleswig-Holstein, among (associate) professionals, vocational grammar school classes, classes introduced by females, younger individuals, members of the project team, and classes introduced face-to-face.

**Table 2:** Potential determinants of class level participation rate (*n* = 363 classes)

**Potential determinants**	**Category**	***n* (%) of classes**	**Mean participation rate**	**Coef. (95% CI)**	***p***
**Federal state, *n* (%)**	Baden-Württemberg	41 (11.3%)	48.3%	Reference	
	Mecklenburg-Vorpommern	21 (5.8%)	13.5%	**−0.35 (−0.49; −0.20)**	***p* < 0.001**
	Niedersachsen	104 (28.7%)	39.3%	−0.09 (−0.19; 0.01)	*p* = 0.072
	Nordrhein-Westfalen	62 (17.1%)	34.0%	**−0.14 (−0.25; −0.04)**	***p* = 0.009**
	Schleswig-Holstein	135 (37.2%)	63.0%	**0.15 (0.05; 0.24)**	***p* = 0.003**
**Study group, *n* (%)**	Intervention	179 (49.3%)	47.5%	Reference	
	Control	184 (50.7%)	45.9%	−0.02 (−0.08; 0.05)	*p* = 0.618
**Educational track, *n* (%)**^a,b^	Professionals/technicians and associate professionals	62 (17.1%)	56.7%	Reference	
	Clerical support workers	41 (11.3%)	49.7%	−0.07 (−0.19; 0.05)	*p* = 0.248
	Service and sales workers	53 (14.6%)	36.6%	**−0.20 (−0.31; −0.09)**	***p* < 0.001**
	Craft related trades workers	67 (18.5%)	41.8%	**−0.15 (−0.25; −0.04)**	***p* = 0.005**
	Plant and machine operators and assemblers/elementary occupations	12 (3.3%)	32.8%	**−0.24 (−0.42; −0.05)**	***p* = 0.012**
	Vocational grammar school^f^	72 (19.8%)	54.7%	−0.02 (−0.12; 0.08)	*p* = 0.696
	Vocational preparation^g^	45 (12.4%)	42.2%	**−0.15 (−0.26; −0.03)**	***p* = 0.014**
**Year of education, *n* (%)**^a,c^	First year	151 (41.6%)	42.9%	Reference	
	Second year	119 (32.8%)	45.6%	0.03 (−0.05; 0.10)	*p* = 0.486
	Third year	35 (9.6%)	47.4%	0.05 (−0.07; 0.16)	*p* = 0.437
**Students in class, M (SD)**^d^		17.8 (6.6)	-	**0.01 (0.001; 0.01)**	***p* = 0.028**
**Present students, M (SD)**		15.7 (6.5)	-	0.002 (-0.003; 0.01)	*p* = 0.475
**Mode of introduction, *n* (%)**	Digitally/hybrid	157 (43.3%)	45.8%	Reference	
	Face-to-face	179 (49.3%)	53.8%	**0.08 (0.02; 0.14)**	***p* = 0.010**
	Email	27 (7.4%)	5.5%	**−0.40 (−0.52; −0.29)**	***p* < 0.001**
**Time for introduction, *n* (%)**^a,e^	45 min	264 (72.7%)	52.9%	Reference	***p* = 0.003**
	70–90 min	66 (18.2%)	40.8%	**−0.12 (−0.20; −0.04)**	
**Introduction by, *n* (%)**	Members of the project team	238 (65.6%)	55.4%	Reference	
	Schoolteachers	53 (14.6%)	37.6%	**−0.18 (−0.26; −0.09)**	***p* < 0.001**
	Addiction prevention experts	33 (9.1%)	34.7%	**−0.21 (−0.31; −0.11)**	***p* < 0.001**
	School social workers	22 (6.1%)	13.4%	**−0.42 (−0.54; −0.30)**	***p* < 0.001**
	Health education students	17 (4.7%)	20.7%	**−0.35 (−0.49; −0.21)**	***p* < 0.001**
**Gender of IP, *n* (%)**	Male	104 (28.7%)	32.7%	Reference	
	Female	259 (71.4%)	52.4%	**0.20 (0.13; 0.26)**	***p* < 0.001**
**Age of IP, M (SD)**		30.4 (8.3)	-	**−0.01 (−0.01; −0.003)**	***p* < 0.001**
**Tandem introduction, *n* (%)**	No	319 (87.9%)	46.0%	Reference	
	Yes	44 (12.1%)	51.8%	0.06 (−0.04; 0.15)	*p* = 0.248

*IP* introducing person, *M* mean, *SD* standard deviation

Bold coefficients indicate statistical significance at *p* = 0.05.

^a^percentages do not add up to 100 due to missing information

^b^information is missing for *n* = 1 (0.3%) class, *n* = 3 (0.8%) classes included students from different educational tracks and *n* = 7 (1.9%) classes included students from different vocational trainings

^c^information is missing for *n* = 33 (9.1%) classes and *n* = 25 (6.9%) classes included students from different years of education

^d^information is missing for *n* = 1 (0.3%) class

^e^information is missing for *n* = 6 (1.7%) of classes and *n* = 27 (7.4%) classes were introduced by email

^f^in Germany, most vocational schools also offer participation in vocational grammar school classes (typically grades 11 to 13) to prepare students for general university entrance certification

^g^these include vocational preparation classes as well as 1- or 2-year basic training with intermediate secondary school-leaving certificate (without training qualification)

### Potential Determinants of Individual Level Participation

To analyze potential determinants of individual participation, we analyzed data of app users (*n* = 4225). Those under the age of 16 (*n* = 10) and those with the incorrect app version (*n* = 307) were excluded. Further, users that requested data deletion (*n* = 17) or did not complete screening (*n* = 580) were excluded due to a lack of baseline data to be analyzed as potential determinants. Thus, the analyses were based on 3311 students (2568 participants; 743 declined). Regarding individual level determinants, many aligned with class level results, but some, like the introduction mode and introducer characteristics, were less prominent or statistically insignificant (Online Resource 6). In terms of individual level variables, being female (vs. male), lower social competencies, cannabis lifetime consumption (vs. never), higher problematic internet use, and higher perceived stress were significant associated with higher participation. ICC was 16.1%, indicating a moderate correlation of individual participation within the same class.

## Discussion

Based on a large sample of German vocational students, the current study aimed to describe the implementation and reach of the newly developed digital prevention program “ready4life.” In terms of implementation, a large flexibility in adjusting to the COVID-19 pandemic was necessary to ensure reaching the goals of introducing over 5000 vocational students and involving 2500 randomized participants (Schmidt et al., 2023).

While the pandemic posed obstacles to school-level participation, individual engagement remained acceptable with a 72% app download rate and 47% participation. Comparable proactive programs in (vocational) school settings achieved up to 80% participation (Haug & Castro, 2018; Haug et al., 2015), but these were conducted pre-pandemic through face-to-face introductions. A parallel Swiss study to evaluate the “ready4life” app (Haug et al., 2022; Paz Castro et al., 2021) reported a similar participation rate (58%) during the pandemic. Our study required digital or hybrid introductions for 43% of sessions, sustaining moderate participation. However, email invitations yielded only a 5% participation rate, underscoring the importance of a proactive, personal approach to program implementation. Another German multi-behavior intervention study within German vocational students supports the feasibility of digital introductions during the pandemic; however, no participation rates were reported (Pietsch et al., 2023).

Class participation rates varied by factors such as federal state, educational track, class size, introduction mode, and introducer characteristics. Higher participation in the state Schleswig-Holstein and when conducted by project team members might be attributable to higher practice and commitment. Higher participation among those from professional trainings or vocational grammar school reflects typical effects of educational differences, as those with lower socio-economic status (e.g., in terms of education, occupation) are typically less likely to participate in prevention efforts (Oliver et al., 2008). Younger introducers possibly acted as role models, enhancing participation. Longer introductions did not yield higher participation, suggesting that concise sessions might be as effective.

Aligned with the intervention’s objectives, study participation at the individual level was more likely for students exhibiting higher perceived stress, lower social competencies, lifetime cannabis consumption, and elevated problematic internet use. These results align with previous studies on digital addiction prevention within (vocational) schools (Haug et al., 2023b; Haug & Castro, 2018; Haug et al., 2015; Paz Castro et al., 2021). Furthermore, females were more likely to participate. Again, this is a typical finding in many prevention areas that males are less likely to participate than females (Oliver et al., 2008). Lower individual participation among first and third year students compared to second year students may be due to the increased awareness and maturity of second year students, while third year students may experience time constraints and shifting priorities as they near the end of their education.

Strengths encompass the participatory app development and high participation despite pandemic constraints. However, the data’s non-representative nature and reliance on self-reporting are limitations. Individual consent after randomization introduces potential selection bias (Puffer et al., 2003). Individual level analyses were conducted solely for students who downloaded the app and completed the app-based screening. The use of raffles may have introduced extrinsic motivation, potentially increasing student engagement and participation beyond what occurs in real-world conditions. The lack of a digital coach outside of the binary gender definition may have lowered interest in participation among those that selected “other” gender. The requirement to have a mobile phone with internet access to participate may have reduced implementation equity based on socio-economic status. Lastly, it cannot be excluded that the specific circumstances and stressors related to the pandemic may have impacted students’ substance use, internet use, or intentions to participate.

The potential implications of the current study are many. First, it highlights the resilience of public health initiatives, particularly in their ability to adapt to unforeseen challenges such as those posed by the COVID-19 pandemic. Secondly, it highlights the critical importance of flexibility in research methods at such times. In addition, the study provides valuable insights into effective recruitment strategies in vocational settings, demonstrating the effectiveness of proactive approaches in engaging the intended target group. Nevertheless, there remains a notable gap in outreach efforts, particularly towards groups such as males or students from non-professional backgrounds. To address this gap, it is imperative that these under-represented groups are involved in both the development and implementation of interventions, thereby ensuring that their perspectives and needs are adequately addressed in future public health initiatives.

## Conclusion

We demonstrated that proactive recruitment via video conferences was effective during the pandemic, with negligible declines in participation rates compared to face-to-face recruitment. In terms of cannabis use, internet use, and life skills, the app successfully resonated with its intended audience. Tailored recruitment and content strategies can enhance engagement, especially among under-represented groups like males and students from non-professional backgrounds. Future research should prioritize their involvement in intervention development to address lower participation rates effectively.

### Supplementary Information

Below is the link to the electronic supplementary material.

Supplementary file1 (PDF 74 KB)

Supplementary file2 (PDF 203 KB)

Supplementary file3 (PDF 83 KB)

Supplementary file4 (PDF 45 KB)

Supplementary file5 (PDF 15 KB)

Supplementary file6 (PDF 49 KB)

## Data Availability

The data that support the findings of this study are available from the corresponding author, D Guertler, upon reasonable request.
