# Supplementary material for: App-Based Addiction Prevention at German Vocational Schools: Implementation and Reach for a Cluster-Randomized Controlled Trial
Source: Prev Sci. 2024 Jul 3;25(5):849–60. doi: 10.1007/s11121-024-01702-w (PMC11322396; doi:10.1007/s11121-024-01702-w)
Supplement: Supplementary file 1 — Supplementary file1 (PDF 74 KB) [file 11121_2024_1702_MOESM1_ESM.pdf]

Online Resource 1 for:

App-based Addiction Prevention at German vocational Schools: Implementation and Reach for a cluster-randomized controlled Trial, Prevention Science

Diana Guertler, Dominic Bläsing, Anne Moehring, Christian Meyer, Dominique Brandt, Hannah Schmidt, Florian Rehbein, Merten Neumann, Arne Dreißigacker, Anja Bischof, Gallus Bischof, Svenja Sürig, Lisa Hohls, Maximilian Hagspiel, Susanne Wurm, Severin Haug, Hans-Jürgen Rumpf

Corresponding author: Diana Guertler, Institute for Community Medicine, University Medicine Greifswald, Walther-Rathenau-Str. 48, 17475 Greifswald, Germany, Phone: +4903834-867765, Fax: 03834/867701, email: diana.guertler@med.uni-greifswald.de

Online Resource 1

Adjustments due to the Covid-19 pandemic

Introduction procedure

Initially, the plan was to train teachers and prevention specialists so that they could integrate the study into existing prevention programs or present it in-person in classrooms using the prepared introductory session. For that purpose, contact was established with several prevention specialists through the Lower Saxony State Office for Addiction Issues who showed interest in the app-based prevention approach. In web conferences, the study and the "ready4life" app were presented, and the prevention specialists were briefed on a possible procedure for implementation.

However, when the pandemic began, teaching was temporarily suspended or replaced by digital instructions, and teachers reported not having the capacity to engage in prevention activities. At the same time, prevention specialists were unable to present the study and the prevention program in vocational schools as planned because either in-person teaching was prohibited, or external individuals were not allowed on school premises. Accordingly, adjustments had to be made to the procedure for recruiting study participants.

The standard operating procedures (SOPs) for the introductory session were modified and adapted for possible digital implementation. However, it was found that both schools and prevention organizations often lacked sufficient technology or suitable platforms for video conferences. As a result, the project team recorded short video clips as an alternative option to introduce the prevention program and the study. These were made available on a video platform, and the link, along with a brief description, was distributed to the students via email.

Several teachers reported that even during regular face-to-face instruction, they did not have the capacity to engage with the prevention program. Therefore, there was an increased demand for an introduction by the project team. At one location, students from healthcare-related training occupations were trained to conduct the introductions themselves, due to their high interest in the topic. Overall, the introductory sessions were offered in both in-person and digital formats (all participants in a video conference) or hybrid formats (the class present on-site with project staff connected via video conference) or via email.

Informed consent procedure

Initially, it was planned to obtain written consent declarations from the students in addition to obtaining consent within the app. However, this proved challenging due to the independent implementation by teachers or digital introductory sessions. In many cases, acronyms were not entered on the form, making them unassignable. Implementation during digital program introductions proved to be entirely impractical. Instead, the procedure for digital consent to participate in the study was improved to allow for the omission of written consent declarations. For underaged students, the EU General Data Protection Regulation does not require the explicit consent of their parents or legal guardians. However, underaged participants were advised that their parents or legal guardians must be informed about study participation. For this purpose, a link to a digital information letter was provided that could be sent directly to parents or legal guardians. Changes to the informed consent procedure were approved by the ethics committee.
