# Supplementary material for: App-Based Addiction Prevention at German Vocational Schools: Implementation and Reach for a Cluster-Randomized Controlled Trial
Source: Prev Sci. 2024 Jul 3;25(5):849–60. doi: 10.1007/s11121-024-01702-w (PMC11322396; doi:10.1007/s11121-024-01702-w)
Supplement: Supplementary file 3 — Supplementary file3 (PDF 83 KB) [file 11121_2024_1702_MOESM3_ESM.pdf]

Online Resource 3 for:

App-based Addiction Prevention at German vocational Schools: Implementation and Reach for a cluster-randomized controlled Trial, Prevention Science

Diana Guertler, Dominic Bläsing, Anne Moehring, Christian Meyer, Dominique Brandt, Hannah Schmidt, Florian Rehbein, Merten Neumann, Arne Dreißigacker, Anja Bischof, Gallus Bischof, Svenja Sürig, Lisa Hohls, Maximilian Hagspiel, Susanne Wurm, Severin Haug, Hans-Jürgen Rumpf

Corresponding author: Diana Guertler, Institute for Community Medicine, University Medicine Greifswald, Walther-Rathenau-Str. 48, 17475 Greifswald, Germany, Phone: +4903834-867765, Fax: 03834/867701, email: diana.guertler@med.uni-greifswald.de

Online Resource 3

Topic specific thresholds for individualised risk and competence profile

	Traffic light feedback		
**Topic and classification criteria**	**Green**	**Yellow**	**Red**
**Stress**<br>Perceived stress<br>(1 "not at all" to 5 "very strongly")	1 or 2	3	4 or 5
**Social competencies**<br>Feeling secure in social situations, mean<br>(1 “very uncertain” to 5 “very certain”)	$\geq$ 3.5	> 2.5 and < 3.5	$\leq$ 2.5
**Social Media/ Gaming**<br>Problematic internet use, sum (0 to 20)	$\leq$ 6	$\geq$ 7 and $\leq$ 8	$\geq$ 9
**Tobacco**<br>Tobacco smoking or nicotine product use in the last 30 days	“never”	“occasionally but not daily”	“(almost) daily“
**Cannabis**<br>Lifetime and last six months consumption of THC-containing cannabis	“no, never” in lifetime or “not at all” within the last six months	“once a month or less“ or “2-4 times a month“ within the last six months	“2-3 times a week“, or “4 times a week or more“ within the last six months

**Alcohol**<br>Consumption days within the past months, Number of standard drinks per drinking day, Maximum number of standard drinks per occasion, Number of standard drinks per months	**Males ≥ 18 years**		
	consumption days ≤ 20 and drinks per drinking day ≤ 2 and maximum number of drinks ≤ 2	(consumption days > 20 or drinks per drinking day > 2 or maximum number of drinks > 2) and drinks per month ≤ 40 and maximum number of drinks ≤ 5	drinks per month > 40 or maximum number of drinks > 5
	**Males aged 16 and 17 years**		
	consumption days ≤ 10 and drinks per drinking day ≤ 2 and maximum number of drinks ≤ 2	(consumption days > 10 or drinks per drinking day > 2 or maximum number of drinks > 2) and drinks per month ≤ 20 and maximum number of drinks ≤ 5	drinks per month > 20 or maximum number of drinks > 5
	**Females ≥ 18 years**		
	consumption days ≤ 20 and drinks drinking per day ≤ 1 and maximum number of drinks ≤ 1	(consumption days > 20 or drinks per drinking day > 1 or maximum number of drinks > 1) and drinks per month ≤ 20 and maximum number of drinks ≤ 4	drinks per month > 20 or maximum number of drinks > 4
	**Females aged 16 and 17 years**		
	consumption days ≤ 10 and drinks per drinking day ≤ 1 and maximum number of drinks ≤ 1	(consumption days > 10 or drinks per drinking day > 1 or maximum number of drinks > 1) and drinks per month ≤ 10 and maximum number of drinks ≤ 4	drinks per month > 10 or maximum number of drinks > 4
