# Supplementary material for: App-Based Addiction Prevention at German Vocational Schools: Implementation and Reach for a Cluster-Randomized Controlled Trial
Source: Prev Sci. 2024 Jul 3;25(5):849–60. doi: 10.1007/s11121-024-01702-w (PMC11322396; doi:10.1007/s11121-024-01702-w)
Supplement: Supplementary file 4 — Supplementary file4 (PDF 45 KB) [file 11121_2024_1702_MOESM4_ESM.pdf]

Online Resource 4 for:

App-based Addiction Prevention at German vocational Schools: Implementation and Reach for a cluster-randomized controlled Trial, Prevention Science

Diana Guertler, Dominic Bläsing, Anne Moehring, Christian Meyer, Dominique Brandt, Hannah Schmidt, Florian Rehbein, Merten Neumann, Arne Dreißigacker, Anja Bischof, Gallus Bischof, Svenja Sürig, Lisa Hohls, Maximilian Hagspiel, Susanne Wurm, Severin Haug, Hans-Jürgen Rumpf

Corresponding author: Diana Guertler, Institute for Community Medicine, University Medicine Greifswald, Walther-Rathenau-Str. 48, 17475 Greifswald, Germany, Phone: +4903834-867765, Fax: 03834/867701, email: diana.guertler@med.uni-greifswald.de

Online Resource 4

Categorisation of occupations into ISCO-08 Major Groups

**ISCO-8 Major Groups**	**Included Occupations**
1 Managers	Not present in the sample
2 Professionals	• Applications programmers<br>• Early childhood educators
3 Technicians and associate professionals	• Administrative and executive secretaries<br>• Chemical and physical science technicians<br>• Civil engineering technicians<br>• Dental assistants and therapist<br>• Draughts persons<br>• Information and communications technology user support technicians<br>• Legal secretaries<br>• Medical and dental prosthetic technicians<br>• Nursing associate professionals<br>• Physical and engineering science technicians<br>• Social work associate professionals
4 Clerical support workers	• Accounting and bookkeeping clerks<br>• Bank tellers and related clerks<br>• Clerical support workers<br>• Contact centre information clerks<br>• General office clerks<br>• Hotel receptionist

	● Secretaries<br>● Stock clerks<br>● Transport clerks
5 Service and sales workers	● Cooks<br>● Domestic housekeepers and housekeeping supervisors in office, hotels and other establishments<br>● Hairdressers<br>● Home-based personal care workers<br>● Shop sales assistants<br>● Waiters
6 Skilled agricultural, forestry and fishery workers	Not present in the sample
7 Craft related trades workers	● Agricultural and industrial machinery mechanics and repairers<br>● Bicycle and related repairers<br>● Bricklayers and related workers<br>● Building and related electricians<br>● Cabinet-makers and related workers<br>● Carpenters and joiners<br>● Electrical mechanics and fitters<br>● Electronics mechanics and servicers<br>● Information and communications technology installers and servicers<br>● Motor vehicle mechanics and repairers

	• Painters and related workers<br>• Plumber and pipe fitters<br>• Pre-press technicians<br>• Precision-instrument makers and repairers<br>• Print finishing and binding workers<br>• Printers<br>• Roofers<br>• Structural metal preparers and erectors<br>• Toolmakers and related workers
8 Plant and Machine Operators and Assemblers	• Chemical products plant and machine operators<br>• Earthmoving and related plant operators<br>• Food and related products machine operators<br>• Heavy truck and lorry drivers<br>• Plastic products machine operators<br>• Paper products machine operators<br>• Rubber products machine operators<br>• Plastic products machine operators<br>• Stationary plant and machine operators
9 Elementary occupations	• Cleaners and helpers in office, hotels and other establishments
0 Armed forces occupations	Not present in the sample
