# Supplementary material for: App-Based Addiction Prevention at German Vocational Schools: Implementation and Reach for a Cluster-Randomized Controlled Trial
Source: Prev Sci. 2024 Jul 3;25(5):849–60. doi: 10.1007/s11121-024-01702-w (PMC11322396; doi:10.1007/s11121-024-01702-w)
Supplement: Supplementary file 5 — Supplementary file5 (PDF 15 KB) [file 11121_2024_1702_MOESM5_ESM.pdf]

Online Resource 5 for:

App-based Addiction Prevention at German vocational Schools: Implementation and Reach for a cluster-randomized controlled Trial, Prevention Science

Diana Guertler, Dominic Bläsing, Anne Moehring, Christian Meyer, Dominique Brandt, Hannah Schmidt, Florian Rehbein, Merten Neumann, Arne Dreißigacker, Anja Bischof, Gallus Bischof, Svenja Sürig, Lisa Hohls, Maximilian Hagspiel, Susanne Wurm, Severin Haug, Hans-Jürgen Rumpf

Corresponding author: Diana Guertler, Institute for Community Medicine, University Medicine Greifswald, Walther-Rathenau-Str. 48, 17475 Greifswald, Germany, Phone: +4903834-867765, Fax: 03834/867701, email: diana.guertler@med.uni-greifswald.de

Online Resource 5

Status of approval of ministries responsible for education and cultural affairs

**Federal state(s)**	**Status of approval**
Baden-Württemberg, Mecklenburg-Vorpommern, Niedersachsen, Schleswig-Holstein	Approval has been granted.
Nordrhein-Westfalen	Approval not necessary for the PARI project as schools are allowed to decide on their participation themselves.
Bayern	Application rejected due to participation incentives in the form of a lottery.
Berlin, Brandenburg	Application not submitted, as it would have required a complete list of participating schools beforehand.
Bremen, Hamburg, Sachsen	Application not submitted, as incentivization of study participants (here, in the form of a lottery) is not allowed.
Hessen	Application not submitted as generally no approval for studies involving the use of personal devices.
Rheinland-Pfalz, Saarland, Sachsen-Anhalt, Thüringen	Application not submitted due to anticipated attainment of the planned sample size.
