# Supplementary material for: App-Based Addiction Prevention at German Vocational Schools: Implementation and Reach for a Cluster-Randomized Controlled Trial
Source: Prev Sci. 2024 Jul 3;25(5):849–60. doi: 10.1007/s11121-024-01702-w (PMC11322396; doi:10.1007/s11121-024-01702-w)
Supplement: Supplementary file 6 — Supplementary file6 (PDF 49 KB) [file 11121_2024_1702_MOESM6_ESM.pdf]

Online Resource 6 for:

App-based Addiction Prevention at German vocational Schools: Implementation and Reach for a cluster-randomized controlled Trial, Prevention Science

Diana Guertler, Dominic Bläsing, Anne Moehring, Christian Meyer, Dominique Brandt, Hannah Schmidt, Florian Rehbein, Merten Neumann, Arne Dreißigacker, Anja Bischof, Gallus Bischof, Svenja Sürig, Lisa Hohls, Maximilian Hagspiel, Susanne Wurm, Severin Haug, Hans-Jürgen Rumpf

Corresponding author: Diana Guertler, Institute for Community Medicine, University Medicine Greifswald, Walther-Rathenau-Str. 48, 17475 Greifswald, Germany, Phone: +4903834-867765, Fax: 03834/867701, email: diana.guertler@med.uni-greifswald.de

Online Resource 6

Sample characteristics of those who downloaded the app and associations with individual level participation (n=3311)

**Potential determinants**	**Category**	**n (%) of students**	**Mean participation rate**	**Odds ratio (95% CI)**	**p**
**Individual Characteristics**					
Gender, n (%)	Males	1,845 (55.7%)	74.9%	Reference	
	Females	1,402 (42.3%)	81.8%	**1.56 (1.28; 1.90)**	**p<0.001**
	Other	64 (1.9%)	62.5%	0.65 (0.37; 1.25)	p=0.141
Age, M (SD) [a]		19.7 (3.9)	-	0.99 (0.97; 1.01)	p=0.335
Number of alcoholic standard drinks per day, Mdn (IQR)		0.2 (0.0-0.9)	-	1.03 (0.98; 1.08)	p=0.235
Number of cigarettes per day, Mdn (IQR) [b]		0.0 (0.0-0.7)	-	1.01 (0.997; 1.03)	p=0.101
Social competencies, M (SD)		29.6 (4.8)	-	**0.98 (0.96; 0.99)**	**p=0.010**
Cannabis consumption lifetime, n (%)	No	1,822 (55.0%)	74.6%	Reference	
	Yes	1,489 (45.0%)	81.2%	**1.47 (1.23; 1.77)**	**p<0.001**
Problematic internet use, M (SD)		9.0 (4.2)	-	**1.04 (1.02; 1.06)**	**p<0.001**
Perceived stress, M (SD)		3.2 (1.2)	-	**1.22 (1.13; 1.31)**	**p<0.001**
General self-efficacy, M (SD)		10.9 (2.1)	-	0.97 (0.93; 1.01)	p=0.143

Class characteristics					
**Federal state, n (%)**	Baden-Württemberg	233 (7.0%)	85.0%	Reference	
	Mecklenburg-Vorpommern	65 (2.0%)	84.6%	0.84 (0.29; 2.43)	p=0.751
	Niedersachsen	881 (26.6%)	73.3%	**0.44 (0.26; 0.76)**	**p=0.003**
	Nordrhein-Westfalen	486 (14.7%)	74.7%	**0.47 (0.26; 0.83)**	**p=0.009**
	Schleswig-Holstein	1,646 (49.7%)	79.3%	0.64 (0.38; 1.07)	p=0.091
**Study group, n (%)**	Intervention	1,647 (49.7%)	78.1%	Reference	
	Control	1,664 (50.3%)	77.0%	0.95 (0.73; 1.22)	p=0.676
**Educational track, n (%)** [c,d]	Professionals / Technicians and associate professionals	656 (19.8%)	79.7%	Reference	
	Clerical support workers	395 (11.9%)	72.7%	**0.62 (0.39; 0.98)**	**p=0.041**
	Service and sales workers	363 (11.0%)	78.8%	0.91 (0.56; 1.46)	p=0.683
	Craft related trades workers	551 (16.6%)	76.6%	0.78 (0.51; 1.19)	p=0.250
	Plant and Machine Operators and Assemblers / Elementary occupations	91 (2.8%)	74.7%	0.67 (0.31; 1.46)	p=0.315
	Vocational grammar school [i]	725 (21.9%)	80.3%	0.97 (0.65; 1.45)	p=0.880
	Vocational preparation [j]	390 (11.8%)	72.3%	**0.57 (0.36; 0.90)**	**p=0.015**
**Year of education, n (%)** [c,e]	First year	1,322 (39.9%)	74.1%	Reference	
	Second year	988 (29.8%)	81.5%	**1.53 (1.14; 2.06)**	**p=0.005**
	Third year	280 (8.5%)	76.4%	1.11 (0.70; 1.76)	p=0.648

**Students in class, M (SD)** [f]		20.5 (6.7)	-	1.003 (0.98; 1.02)	p=0.746
**Present students, M (SD)** [g]		18.3 (6.8)	-	1.01 (0.99; 1.03)	p=0.434
**Mode of introduction, n (%)**	Digitally/hybrid	1,493 (45.1%)	77.2%	Reference	
	Face-to-Face	1,772 (53.5%)	77.9%	1.03 (0.80; 1.33)	p=0.818
	Email	46 (1.4%)	73.9%	0.68 (0.24; 1.96)	p=0.476
**Time for introduction, n (%)** [a,h]	45 Minutes	2,760 (83.4%)	77.7%	Reference	
	70-90 Minutes	481 (14.5%)	78.0%	1.14 (0.80; 1.62)	p=0.482
**Introduction by, n (%)**	Members of the project team	2,581 (78.0%)	77.9%	Reference	
	Schoolteachers	267 (8.1%)	77.9%	1.02 (0.67; 1.56)	p=0.919
	Addiction prevention experts	263 (7.9%)	83.7%	1.50 (0.91; 2.48)	p=0.110
	School social workers	70 (2.1%)	54.3%	**0.32 (0.15; 0.68)**	**p=0.003**
	Health education students	130 (4.0%)	70.8%	0.60 (0.34; 1.04)	p=0.069
**Gender of IP, n (%)**	Male	589 (17.8%)	77.4%	Reference	
	Female	2,722 (82.2%)	77.6%	0.99 (0.72; 1.36)	p=0.966
**Age of IP, M (SD)**		28.5 (6.3)	-	1.02 (0.995; 1.04)	p=0.135
**Tandem introduction, n (%)**	No	2,735 (82.6%)	77.7%	Reference	
	Yes	576 (17.4%)	76.9%	0.87 (0.61; 1.23)	p=0.430

Note. IP, introducing person; M, Mean; SD, Standard Deviation; Mdn, Median; IQR, Inter-Quartile-Range; Bold coefficients indicate statistical significance at p=0.05; [a] Information is missing for n=1 (0.03%) participant; [b] Information is missing for n=2 (0.1%) participants; [c] Percentages do not add up to 100 due to missing information; [d] Information is missing for n=19 (0.6%) participants, n=48 (1.5%) students came from classes including different educational tracks, and n=73 (2.2%) students came from classes including different vocational trainings; [e] Information is missing for n=366 (11.1%) students and n=355 (10.7%) students came from classes with different years of education; [f] Information is missing for n=62 (1.9%) students;

[g] Information is missing for n=78 (2.4%) students; [h] Information is missing for n=24 (0.7%) of students and n=46 (1.4%) students came from classes introduced by email; [i] In Germany most vocational schools also offer participation in vocational grammar school classes (typically grades 11 to 13) to prepare students for general university entrance certification; [j] These include vocational preparation classes as well as 1- or 2-year basic training with intermediate secondary school-leaving certificate (without training qualification).
